# Immediate post‐diagnostic support for people with dementia and thier care partners: Scoping literature review

**DOI:** 10.1002/alz.084429

**Published:** 2025-01-09

**Authors:** Sladana Pavkovic, Lyn R. Goldberg, Jane E Alty, Melissa Abela, Lee‐Fay Low

**Affiliations:** ^1^ Wicking Dementia Research and Education Centre, University of Tasmania, Hobart, TAS Australia; ^2^ Wicking Dementia Research and Education Center, University of Tasmania, Hobart, TAS Australia; ^3^ Royal Hobart Hospital, Hobart, TAS Australia; ^4^ The University of Sydney, Sydney, NSW Australia

## Abstract

**Background:**

Timely post‐diagnostic support is necessary to assist people living with dementia and their families to preserve their independence and to facilitate emotional and practical adaptation to dementia diagnosis and life after diagnosis. However, there is a noticeable scarcity of evidence concerning support following dementia diagnosis.

Our objective was to examine the following aspects: the nature and type of evidence pertaining to post‐diagnostic support within the initial two years of diagnosis; the conceptualization of post‐diagnostic support; and the existing gaps in the literature concerning this essential support.

**Method:**

We conducted a comprehensive scoping literature review following the systematic process outlined by the Joanna Briggs Institute (JBI), employing the Population‐Who, Concept‐What, Context‐With What Qualifiers (PCC) protocol. All relevant keywords and index terms were systematically searched across PubMed, Scopus, CINAHL, and PsychInfo databases. The review encompassed evidence spanning from 2005 to 2023. The screening process is visually represented in the PRISMA diagram. Subsequently, two authors independently applied critical appraisal to the studies using the Mixed Method Appraisal Tool.

**Result:**

For eligibility, a total of 506 articles were assessed, leading to the inclusion of 29 papers derived from 26 studies characterized by high methodological quality. Notably, the majority of these articles (n = 14, from 12 studies) were authored by researchers based in the UK. High methodological quality was reflected in various types of evidence, encompassing reviews (n = 2), mixed studies (n = 8), quantitative studies (n = 9), and qualitative studies (n = 7).

Specialist memory clinic support emerged as the most prevalent form of assistance during the initial two years following a dementia diagnosis. This support varied in its implementation, with some studies indicating support provided in (n = 12) or by (n = 5) memory clinics. Additionally, other studies presented post‐diagnostic support through diverse avenues, including day centres, on‐going support workers; home support programs, and community or non‐government organization initiatives.

**Conclusion:**

A notable literature gap exists in immediate post‐diagnostic support for people with dementia and care partners. However, it remains unclear whether this gap is confined to the literature alone or extends to actual practice. Further research is imperative to delve into the support programs following dementia diagnosis, with a specific emphasis on settings beyond memory clinics.

